# Quantitative relationships between national cultures and the increase in cases of novel coronavirus pneumonia

**DOI:** 10.1038/s41598-023-28980-8

**Published:** 2023-01-30

**Authors:** Ningyao Yu, Le Tao, Guilin Zou

**Affiliations:** College of Marxism, Xiangtan University, Xiangtan, 411105 Hunan China

**Keywords:** Scientific data, Statistics, Diseases

## Abstract

Support vector machine (SVM) and genetic algorithm were successfully used to predict the changes in the prevalence rate (ΔPR) measured by the increase of reported cases per million population from the 16th to the 45th day during a nation’s lockdown after the COVID-19 outbreak. The national cultural indices [individualism–collectivism (Ind), tightness–looseness (Tight)], and the number of people per square kilometer (Pop_density) were used to develop the SVM model of lnΔPR. The SVM model has R^2^ of 0.804 for the training set (44 samples) and 0.853 for the test set (11 samples), which were much higher than those (0.416 and 0.593) of the multiple linear regression model. The statistical results indicate that there are nonlinear relationships between lnΔPR and Tight, Ind, and Pop_density. It is feasible to build the model for lnΔPR with SVM algorithm. The results suggested that the risk of COVID-19 epidemic spread will be reduced if a nation implements severe measures to strengthen the tightness of national culture and individuals realize the importance of collectivism.

## Introduction

According to the data released by WHO (https://www.who.int/), the number of confirmed cases of COVID-19 worldwide reached 562 672 324, of which 6 367 793 died, since the outbreak of COVID-19 in Wuhan in December 2019 to 21 July 2022 at 00:17 am GMT+8. The COVID-19 pandemic has seriously threatened people's lives and health, and brought about serious impacts on global societies, economies and politics^1^. In the face of the unprecedented global public health crisis of COVID-19, governments with different national cultures will adopt different response measures, resulting in different epidemic prevention effects. The establishment of prediction models for COVID-19 prevalence rate is of great significance for assisting the government to develop a more effective pandemic prevention measures that are based on its own national cultural characteristics.

Since the outbreak of COVID-19, some researchers have used mathematical models to explore the spread models about COVID-19^2,3^. These models can be roughly divided into two categories, propagation dynamics models and phenomenological models^4^. These studies that belong to the propagation dynamics models include susceptible-infected-recovered (SIR) models^5–7^, conceptual mathematical model derived from the susceptible-exposed-infected- recovered (SEIR) isolation models^8^, dynamic model of nonlinear equations based on fractional order^9^. Most transmission dynamics models rely heavily on data and parameters that are difficult to obtain precisely, such as population movements, behavioral habits, the basic number of regenerations R_0_, the number of current infections, the mortality rate of infections, or the proportion of asymptomatic (infectious) individuals. In general, any small change in these input data can result in models producing a prediction bias of several tens of times^4^. The phenomenological models require relatively few parameters, such as environmental risk to which the city or region is exposed^10,11^, population density, temperature^12^, and absolute humidity^13^, lockdown time, gross domestic product^14^ or GDP per capita, health care expenditure, air pollution levels^15^. Recently, Haouari and Mhir^16^ proposed a phenomenological model, which iteratively solved the quadratic programming problem using the particle swarm optimization method to predict the number of deaths from the COVID-19. The input data on which the model relies are easy to obtain precisely.

Besides the above factors, national culture is an important factor affecting the effectiveness of COVID-19 epidemic prevention and control measures. In the face of the global spread of the epidemic, governments have implemented various interventions to mitigate or even stop the spread of COVID-19, including strict lockdowns of cities, regions or borders, home isolation, tracking and isolating COVID-19 patients, observing social distancing, mask mandates, frequent hand washing, etc. The implementation of these measures requires not only the firm determination of governments at all levels, but also the conscious compliance of the public. Culture is a system of behavioral norms that reflect how people behave, and citizens with different cultures may respond differently to the same challenge^17^. The group of Cao^18^ used hierarchical regression to detailly analyze the relationships between the efficiency of COVID-19 anti-epidemic measures and national tightness–looseness (Tight), individualism–collectivism (Ind), patient median age, the number of people per square kilometer (Pop_density) and other indices. The contributions of this study are:

- Utilize support vector machine (SVM) algorithm to establish a quantitative relationship model between national culture indices and increased cases of novel coronavirus pneumonia, measured by the changes in the prevalence rate (ΔPR): the increase of reported cases per million population from the 16th to the 45th day during a nation’s lockdown after the COVID-19 outbreak.
- Predict the increase in cases of novel coronavirus pneumonia in a country (or region), for assisting governments to develop more effective prevention and control measures based on their own national cultural characteristics to curb the spread of COVID-19.

## Methods

The SVM algorithm is a supervised learning method that can be applied to classification (or regression) tasks. Since the SVM appeared in the late 1960s, this technology has been deeply improved and applied in many different fields. SVMs are called maximum interval classifiers because they find the optimal hyperplane determined by many support vectors between the two classes. Their excellent generalization ability is attributed to the introduction of penalty factor C, which allows a certain number of misclassifications to prevent the effects of outliers.

The SVM algorithm initially provided only linear classification. Due to the introduction of kernel functions, SVMs can also deal with nonlinear problems. The principle is to use kernel functions to map data points to high-dimensional feature spaces, and then carry out linear classification or linear regression. Thus, each dot product is replaced by a nonlinear kernel function, allowing the technique to find the largest spaced hyperplane in transformed high-dimensional spaces. There are many kernel functions available, among which the radial basis function (RBF)-based method is widely used.

SVM algorithms based on RBF kernel function have two parameters (penalty factor C and width coefficient γ) that must be optimized because they affect the predictive performance of a model. The parameter C is a regularization parameter, which controls the trade-off between achieving a low error on the training data and minimizing the norm of the weight. If the value is too larger, it increases the penalty for inseparable points, resulting in the use of too many support vectors and overfitting. Conversely, if C is too smaller, there may be a poor fit. Similar to C, the γ parameter describing the width of the RBF kernel results in overfit or underfit for the model when the γ value is too larger or too smaller^19,20^.

**Figure 1:**
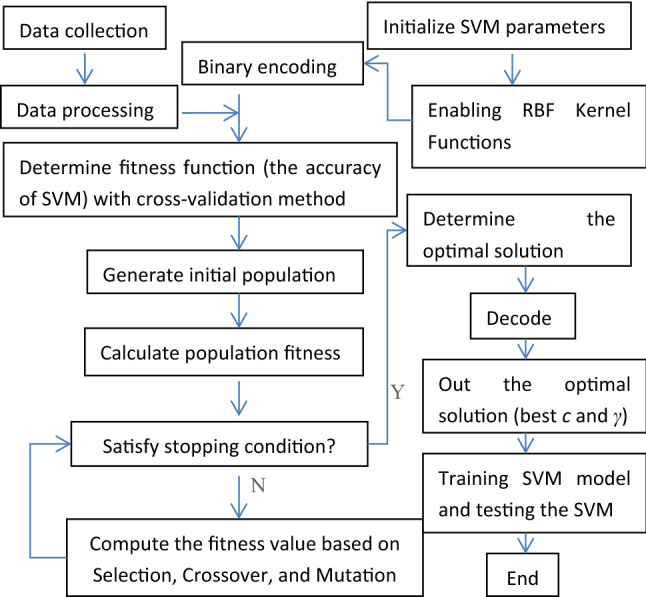
Flow chart of SVM SVM based on genetic algorithm.

Usually, genetic algorithm (GA) and particle swarm optimization (PSO) algorithms can be used in C and γ optimization. The former is superior to the latter in the optimization speed, so genetic algorithm was used to find the best C and γ parameters in this paper. This algorithm mimics the process of natural evolution and genetics, where the process of mutation, selection, and crossover results in the fittest individuals surviving. Figure 1 shows the flow chart of SVM based on genetic algorithm^21^. Firstly, the problem to be solved is encoded as a chromosome or individual in the genetic space; Then the genetic manipulation steps are followed that include (1) selection of the winning individual from the population, (2) crossover by choosing a random position on the binary string and exchanging the segments with another string partitioned similarly, and (3) variation (a small probability of mutation randomly changing certain genetic of individuals in the population). Finally, the optimization process stops when the fitness of the optimal individual reaches a given threshold or the number of iterations reaches a preset number. The candidate solutions of the next generation (individual or chromosome) are superior to the previous generation because they inherit the excellent traits of the previous generation' solutions. That is to say, the candidate solutions evolve in the direction of optimal solutions. In optimization, genetic algorithm parameters such as the maximum number of generations, the maximum population size, the crossover probability, and the mutation probability need to be set in advance^20^.

The data in Table 1 is derived from the literature^18^, where increased cases of novel coronavirus pneumonia (ΔPR) are measured by the increase of reported cases per million population from the 16th to the 45th day during a nation’s lockdown after the COVID-19 outbreak. Tight is a national culture index of tightness–looseness. The culture index Ind denotes the individualism–collectivism. Pop_ density is the number of people per square kilometer. The ΔPR values are serious uneven distribution, e.g., the maximum value (Singapore ΔPR = 3536) is 7072 times of the lowest value (Vietnam ΔPR = 0.5). Thus, ΔPR values are converted to the natural logarithmic form, lnΔPR, in this paper.

**Table 1:** Increased cases of novel coronavirus pneumonia in 55 countries and parameters used.

No	Country	Tight	Ind	Pop_ density	lnΔPR/ reported	lnΔPR/SVM	lnΔPR/MLR
Training set							
1	Japan	42.8	46	347.8	3.68	3.67	5.37
2	Belgium	98.1	75	375.6	8.01	8.00	7.67
3	Argentina	73.4	46	16.2	4.62	5.41	5.91
4	Netherlands	62.6	80	508.5	7.36	7.35	7.10
5	Portugal	67.3	27	112.4	7.37	5.71	5.14
6	Austria	71.6	55	106.7	6.46	6.47	6.23
7	Lithuania	46.1	60	45.1	4.10	6.63	5.80
8	Venezuela	46.0	12	36.3	1.95	4.68	4.08
9	India	81.9	48	450.4	3.67	3.68	6.37
10	Indonesian	3.0	14	145.7	3.63	3.62	3.23
11	Vietnam	30.7	20	308.1	−0.69	−0.68	4.15
12	Iran	23.6	41	49.8	6.86	5.43	4.62
13	Singapore	42.2	20	7915.7	8.17	8.16	7.93
14	China	38.2	20	147.7	3.54	3.55	4.24
15	Luxembourg	94.7	60	231.4	7.77	7.76	6.99
16	South Korea	29.7	18	528.0	4.07	4.08	4.16
17	South Africa	71.5	65	46.8	4.84	5.58	6.56
18	Hungary	43.7	80	108.0	5.28	5.29	6.49
19	United Kingdom	74.5	89	272.9	7.71	7.70	7.58
20	Jordan	3.9	30	109.3	2.87	4.34	3.80
21	Pakistan	7.0	14	255.6	4.59	4.58	3.37
22	Canada	77.0	80	4.0	7.02	7.01	7.20
23	Morocco	6.1	46	80.1	4.69	4.70	4.41
24	Chile	82.0	23	24.3	6.67	6.66	5.29
25	Estonia	51.3	60	31.0	6.72	6.71	5.91
26	Egypt	2.6	25	98.0	3.97	3.96	3.59
27	Bangladesh	3.2	20	1265.0	4.40	4.41	3.97
28	Saudi Arabia	20.4	25	15.3	5.86	5.87	3.96
29	Tanzania	23.3	25	64.7	2.08	3.51	4.05
30	Mexico	80.7	30	66.4	5.18	5.19	5.53
31	Slovenia	74.6	27	102.6	5.84	5.85	5.30
32	Nigeria	19.4	30	209.6	3.11	3.12	4.20
33	Ireland	62.1	70	69.9	8.01	6.62	6.53
34	Turkey	21.5	37	104.9	6.39	5.32	4.45
35	Bulgaria	60.5	30	65.2	4.98	4.97	5.07
36	Peru	47.8	16	25.1	6.91	6.05	4.26
37	Slovakia	65.8	52	113.1	5.26	6.11	5.99
38	Greece	61.8	35	83.5	4.47	5.64	5.28
39	Iceland	42.4	60	3.4	7.73	7.74	5.70
40	Poland	50.9	60	124.0	5.60	5.59	5.95
41	Denmark	60.2	74	136.5	7.03	7.02	6.66
42	Finland	66.2	63	18.1	6.36	6.10	6.35
43	Ukraine	59.0	25	77.4	5.42	5.30	4.86
44	Spain	80.6	51	93.1	8.01	6.41	6.28
Test set							
45	Philippines	39.3	32	351.9	4.09	2.62	4.79
46	Czech Rep	58.8	58	137.2	6.06	5.78	6.06
47	Germany	71.8	67	237.0	7.00	7.59	6.72
48	Italy	66.4	76	205.9	7.62	9.28	6.91
49	Russia	53.6	39	8.8	7.34	7.03	5.21
50	France	82.8	71	122.6	7.08	8.31	7.06
51	United States	60.8	91	35.6	7.89	8.68	7.24
52	Albania	35.0	20	104.9	5.23	5.87	4.15
53	Sweden	61.2	71	24.7	7.31	6.93	6.53
54	Romania	46.0	30	85.1	6.26	5.53	4.75
55	Croatia	53.6	33	73.7	5.61	5.23	5.02

## Results and discussions

By using SPSS 19.0 software, stepwise multiple linear regression (MLR) analysis was performed for the increase in cases of novel coronavirus pneumonia (lnΔPR) and the individualism–collectivism (Ind), tightness–looseness (Tight), and the number of people per square kilometer (Pop_density), national policy severity index Govt_Strgcy, and median age Median_age^18^. A regression equation containing three variables (Tight, Ind, and Pop_ density) was obtained. The data set was randomly divided into a training set with 44 samples (80%) and a test set having 11 samples (20%), which are listed in Table 1. The training set is used to build a model, and the test set is used to validate the model. Table 2 shows the descriptive statistics of variables. Both of the Skewness and Kurtosis statistic values for Pop_ density are greater than 3, suggesting that its data is seriously abnormal.

**Table 2:** Descriptive statistics.

Variable	*N*	Minimum	Maximum	Mean	Std. Deviation	Skewness	Kurtosis
Tight	55	2.60	98.10	50.9309	24.87817	−0.407	−0.575
Ind	55	12.00	91.00	44.9455	22.44204	0.337	−1.140
Pop_ density	55	3.40	7915.70	297.8291	1064.77005	7.044	51.066
lnΔPR/reported	55	−0.69	8.17	5.6193	1.86034	−0.864	1.004

The coefficients of the linear regression equation obtained are shown in Table 3. The coefficients R^2^ of the training set and the test set are 0.416 and 0.593, respectively. It is generally regarded that a regression model has statistical significance if its coefficient of determination R^2^ is greater than 0.5. But the linear model in this paper has a determination coefficient R^2^ of 0.416 < 0.5, indicating that the MLR model possesses poor statistical results. Therefore, the SVM algorithm was used to improve the statistical quality of the models for the increase in cases of novel coronavirus pneumonia lnΔPR, by applying the LIBSVM toolbox on the MATLAB R2014a platform. During the modeling process, the parameter values in GA were set as follows: the maximum number of generations being 200, the maximum population size being 20. In addition, the default values were adopted for the crossover probability and the mutation probability. The leave-one-out (LOO) method was used for cross-validation in the optimization of SVM parameters (C and γ). This method uses one observation from the training set as the validation data, and the remaining n − 1 (n: the total number of samples in the training set) observations as the training data^22^. This is repeated n times such that each observation in the training set is used once as the validation data. The mean squared error (MSE) from cross-validation was used to evaluate the SVM parameters. The search range of SVM parameter C was 1 ~ 200, and the γ parameter varied from 0 to 0.1. In the end, the optimal parameters of C = 126.0 and γ = 1.80 × 10^–5^ were obtained.

**Table 3:** Coefficients for linear regression equation.

Variable	Coefficient	S.E	t	Sig	VIF
Constant	2.595	0.582	4.462	0.000	–
Tight	0.023	0.010	2.226	0.032	1.352
In	0.036	0.013	2.829	0.007	1.386
Pop_ density	4.63E−4	2.01E−4	2.306	0.026	1.031

**Figure 2:**
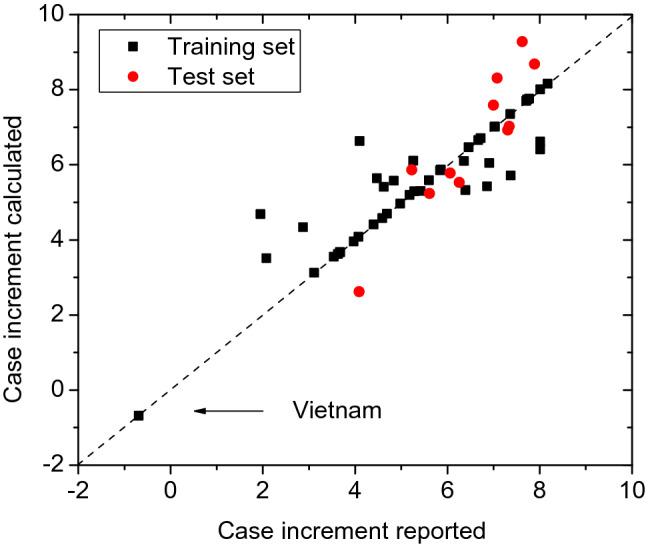
Relationships between the predicted and reported prevalence rates (generated by the OriginPro 8.5.0 SR1 software—http://www.OriginLab.com).

Based on the optimal SVM parameters (C = 126.0; γ = 1.80 × 10^–5^), the 11 samples in the test set were predicted. The prediction results from the optimal SVM model are listed in Table 1 and the statistical results are listed in Table 4. The coefficient of determination R^2^ from the optimal SVM mode are 0.804 for the training set and 0.853 for the test set, which are much higher than the acceptance criteria (R^2^ = 0.5), indicating that the optimal SVM model is statistically significant. For the training set, the SVM model yielded the Akaike information criterion (AIC) value of −7.22, the Bayesian information criterion (BIC) value of −1.86, and the mean absolute percentage error (MAPE) of 11.80%, which are respectively lower than that (39.85, 45.20 and 37.92%) from the MLR model. In addition, the coefficient of determination R^2^ in the optimal SVM mode are much larger than that (0.416 and 0.593, respectively) in the MLR model, indicating that there are nonlinear relationships between the dependent variable lnΔPR and the independent variable (Tight, Ind, Pop_ density). Therefore, it is appropriate to use SVMs to model the increase in cases of novel coronavirus pneumonia. Figure 2 shows the relationships between the predicted prevalence rate lnΔPR and the actual lnΔPR.

**Table 4:** Comparison of model statistics in MLR and SVM models.

Model	Subset	*N*	*R*^2^
MLR	Training set	44	0.416
	Test set	11	0.593
SVM	Training set	44	0.804
	Test set	11	0.853

Table 3 shows that the two national culture indices including individualism–collectivism (Ind), tightness–looseness (Tight), and the number of people per square kilometer (Pop_density) have sig.-values less than 0.05, indicating that all the three parameters are significantly related to the increase in cases of novel coronavirus pneumonia (lnΔPR). In addition, the VIF value of the three parameters is less than five, indicating that there is no serious multicollinearity problem among the three parameters, Ind, Tight, and Pop_density, that is, the three parameters can independently reflect the influencing factors of the increase in cases of novel coronavirus pneumonia.

Model validation from the 11 samples in test set leads to the determination coefficient between the predicted and reported lnΔPR being R^2^ = 0.853; the validation coefficient q_ext_^2^ = 0.673; at intercept = 0, the slopes k′ of regression line (experimental versus predicted lnΔPR) = 0.953 and k (predicted versus experimental lnΔPR) = 1.033; determination coefficient R_0_^′2^ (experimental versus predicted lnΔPR) = 0.922; R_0_^2^ (predicted versus experimental lnΔPR) = 0.768; and r_m_^2^ (= R^2^ × [1 − (R^2^ − R_0_^2^)^1/2^]) = 0.604. The optimal SVM model meets the criteria^23^: q_ext_^2^ > 0.5; R^2^ > 0.6; (R^2^ − R_0_^2^)/R^2^ (= 0.0996) < 0.1; 0.85 ≤ k^′^ ≤ 1.15; and r_m_^2^ > 0.5, which suggests that the optimal SVM model proposed is successful.

Systematic bias test was further carried out for the optimal SVM model. A regression model should not meet any one of the following conditions, otherwise it possesses systematic bias in predictions^24^: (1) NPE/NNE > 5 or NNE/NPE > 5 (NPE: the number of positive errors, NNE: the number of negative errors); (2) ABS(MPE/MNE) > 2 or ABS(MNE/MPE) > 2 (ABS: absolute value, MPE: the mean positive error, MNE: the mean negative error); (3) AAE – ABS(AE) < 0.5 × AAE (AAE: the average absolute error, AE: the average error); (4) R^2^(ith vs (i − 1)th residuals) > 0.5 for residuals sorted on Y_obs_; (5) R^2^ (Y vs. residuals) > 0.5.

The results of systematic bias test are as follow: NPE/NNE = 6/5 < 5; ABS(MPE/MNE) = 0.982/0.592 < 2; AAE − ABS(AE) = 0.769 − 0.124 = 0.645 > 0.5 × 0.769 = 0.385; R^2^(ith vs (i − 1)th residuals) = 0.002 < 0.5 for residuals sorted on Y_obs_; R^2^(Y vs residuals) = 0.390 < 0.5. These results suggest that the optimal SVM model (C = 126.0; γ = 1.80 × 10^–5^) does not meet above circumstances, that is, it has no systematic error in predicting lnΔPR ^24^.

**Figure 3:**
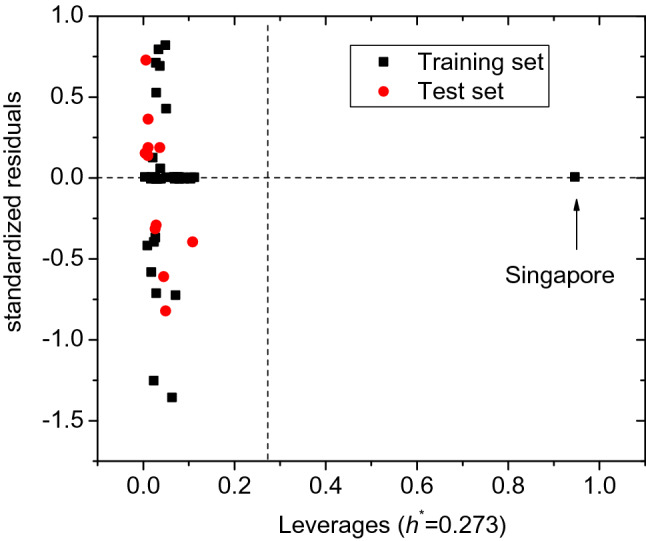
The standardized residuals vs the leverage values (generated by the OriginPro 8.5.0 SR1 software—http://www.OriginLab.com).

Figure 3 shows the Williams plot, which reflects the relationships between the standardized residuals predicted by the optimal SVM model and the leverage values. The Williams plot is used to describe the application domain of the optimal model. The prediction results are reliable only if the prediction points fall into this domain, where the absolute values of the standardized residual are not more than 3 and the leverage values are less than the warning leverage value h^*^. h^*^ is calculated with h^*^ = 3 × (p + 1)/n, where p is the number of parameters and n is the number of samples in the model. Figure 3 shows that only the sample point of Singapore is outside the applicability domain, indicating that the argument sample is an "outlier" compared to other sample variables. But its standardized residuals being close to 0 indicates that the optimal SVM model has a strong predictive power for the increase in cases of novel coronavirus pneumonia lnΔPR ^20^. Similarly, according to the median absolute deviation method^25,26^, the sample point of Vietnam (No. 11) has a determination coefficients D of 3.22, above the threshold of 2.5, and belongs to outliers. However, its lnΔPR value predicted with the SVM model is accurate compared with that from the MLR model.

In this paper, the SVM model of the increase in cases of novel coronavirus pneumonia lnΔPR contains three parameters, individualism–collectivism (Ind), tightness–looseness (Tight), and the number of people per square kilometer (Pop_density). As is obvious, there are correlations between population density Pop_ density and lnΔPR, because an increasing population density, Pop_density, leads to people more likely to be in close contact, and results in higher ΔPR. Thus, this article focuses on the relationships between lnΔPR and the national cultural indices Ind and Tight.

For decades, cultural studies have attracted scholars from a variety of disciplines, including anthropologists, sociologists, and psychologists. Despite its universality, ambiguity, and diversity, it is widely believed that culture encompasses values, beliefs, norms, and other factors that guide and influence human behavior in society^18^. The famous Dutch social psychologist, Geert Hofstede^27,28^ pioneered six dimensions to measure the cultural systems of different countries, of which, the individualism–collectivism culture is the most commonly used one, with the strongest predictive power. The individualism–collectivism index Ind represents the extent to which the individual sees himself primarily as an autonomous entity (individualism) or embedded in a closely connected group (collectivism). Collectivist culture prioritizes the social role of the individual in the group and places it above individual achievement. Conversely, an individualistic culture refers to an individual acting in his or her own interests, without any social affiliation or obligation, and with no intention of protecting the interests of other members^18,29^. The individualism–collectivism culture index Ind varies from 1 to 100 points. The higher the score, the more the national culture is displayed as individualistic. In a society emphasizing collective achievement, people tend to adhere strictly to government restrictive directives and recommendations. Conversely, individualists may be more concerned about the interests of themselves and their immediate family. The higher the Ind value, the more individualistic the nation, which naturally hinders efforts to contain the spread of the virus, resulting in an increase in ΔPR. Therefore, ΔPR is positively correlated with Ind.

Professor Michele Gelfand, a prominent cultural psychologist at the University of Maryland, has studied the national culture index, tightness- looseness^30^ and argued that cultural tightness comes from the strength of social norms and the intensity of sanctions. Cultures in which groups require individuals to adhere to group norms and have less tolerance for behavior that deviates from group norms are defined as tight cultures, while cultures that are less normative and can tolerate individual deviations are defined as loose cultures. The national tightness- looseness culture index Tight can be examined at three levels: (1) microscopic, individual or community level, (2) mesoscopic, regional, city or provincial level, and (3) macroscopic, or national level^18^. Gelfand et al.^31^ based on a survey of nearly 7000 people in 33 countries, asked respondents to assess the veracity of some of their statements, and found that the greater the pressure on a society, whether it is a natural or man-made disaster, the tighter the culture formed by this society. For the first time, they proposed the quantitative description of cultural tightness–looseness, that is, the Gelfand tightness–looseness index. Another cultural psychologist, Irem Uz^32^ developed the Gelfand tightness–looseness index, proposing the Uz tightness–looseness index. This index has an advantage over the Gelfand tightness–looseness index because Uz et al. surveyed a wider scope and more countries. Thus, the Uz tightness–looseness index has been widely used. The lower the value of the national tightness–looseness index Tight, the greater the intensity of social norms and sanctions in the nation's culture, e.g., Japan, Singapore, and China. The larger the Tight, the more loose the national cultural identity (e.g., Italy, the United States, France), which may have a negative effect on government recommendations such as stay-at-home quarantine, mask mandates, observing social distancing, leading to the more increased cases of novel coronavirus pneumonia ΔPR in the nation^33^. Therefore, ΔPR is also positively correlated with Tight.

It should be pointed out that the proposed model possesses a relatively smaller data set, especially the lack of samples from African countries. Collecting more data on ΔPR will make the model more representative.

## Conclusions

By using the national culture tightness–looseness, Tight, individualism–collectivism, Ind, together with the number of people per square kilometer, Pop_ density as independent variables, and the increase in cases of novel coronavirus pneumonia lnΔPR as the dependent variable, quantitative relationship models between the independent variables and the dependent variable were successfully established with the GA and SVM algorithms. The accuracy of the SVM model in predicting the increase in cases of novel coronavirus pneumonia lnΔPR from the 16th to the 45th day during a nation’s lockdown after the COVID-19 outbreak is much greater than that of the linear model. The use of SVM to establish a lnΔPR model is successful. Based on the analysis of the SVM model, the larger the individualism–collectivism Ind index, the more the national culture is displayed as individualism, which is not conducive to curbing the spread of the virus, resulting in an increase in the number of COVID-19 infection cases. The larger the national culture tightness–looseness index Tight, the looser the cultural identity of the nation, which may reduce people's conscious compliance with government instructions and recommendations and reduce the effectiveness of epidemic prevention measures. The results suggested that the risk of COVID-19 epidemic spread will be reduced if a nation implements severe measures to strengthen the tightness of national culture and individuals are aware of the importance of collectivism. In addition, reducing population density can help reduce people's access, thereby reducing the spread of the epidemic.

## Data Availability

All data generated or analysed during this study are included in this published article.
